# Zika preparedness and response in Viet Nam

**DOI:** 10.5365/wpsar.2018.9.1.002

**Published:** 2018-06-14

**Authors:** Dong T Nguyen, Hung T Do, Huy X Le, Nghia T Le, Mai Q Vien, Trieu B Nguyen, Lan T Phan, Thuong V Nguyen, Quang C Luong, Hung C Phan, Hai T Diep, Quang D Pham, Thinh V Nguyen, Loan KT Huynh, Dung CT Nguyen, Hang TT Pham, Khanh KH Ly, Huong NLT Tran, Phu D Tran, Tan Q Dang, Hung Pham, Long N Vu, Anthony Mounts, S Arunmozhi Balajee, Leisha D Nolen

**Affiliations:** a Pasteur Institute, Nha Trang, Viet Nam.; b Pasteur Institute, Ho Chi Minh City, Viet Nam.; c General Department of Preventive Medicine, Hanoi, Viet Nam.; d Division of Global Health Protection, Centers for Disease Control and Prevention, Atlanta, GA.; e Arctic Investigation Program, Division of Preparedness and Emerging Infections, National Center for Emerging and Zoonotic Infectious Diseases, Centers for Disease Control and Prevention, Anchorage, AK.

## Abstract

The findings and conclusions in this article are those of the authors and do not necessarily represent the official positions of the United States Centers for Disease Control and Prevention.

This article describes Viet Nam Ministry of Health’s (VMoH) activities to prepare for and respond to the threat Zika virus (ZIKV), including the adaptation of existing surveillance systems to encompass ZIKV surveillance.

On 1 February 2016, the World Health Organization (WHO) declared the Zika epidemic a Public Health Emergency of International Concern (PHEIC). (*1*) Following this declaration, the VMoH developed a national ZIKV preparedness and response plan that encompassed coordination, prevention, surveillance, care and treatment, communication, logistics and international cooperation. The national emergency operations centre (EOC) at the General Department of Preventive Medicine (GDPM), using Global Health Security Agenda (GHSA) resources, served as the nerve centre for these activities. The GDPM, the central public health agency within the VMoH, created the ZIKV response plan, including training health-care workers to recognize and report ZIKV infection, strengthening surveillance and building ZIKV diagnostic testing capacity. The plan was implemented by the four Vietnamese regional public health institutes (RPHI) that serve as the regional surveillance and laboratory lead for outbreak preparedness and response. A task force comprising epidemiologists, laboratorians, health communications specialists, local government leaders and clinicians was established in each region.

Teams made up of trainers from the GDPM, the Medical Services Administration, the Maternal and Child Health Department, the Health Communication and Education Department and international experts from WHO and the United States Centers for Disease Control and Prevention (CDC) were deployed to each region to train clinicians to recognize and report ZIKV. Training included a training-of-trainers component and was followed by a series of cascading workshops to lower administrative levels. Three ZIKV informational trainings were conducted covering all 63 provinces within the country, educating a total of 637 local health-care providers and authorities. Additionally, guidance for clinicians regarding early diagnosis, services, and care of pregnant women was developed and disseminated to health-care providers.

An existing sentinel surveillance system for the dengue virus was expanded in eight southern provinces to include ZIKV, as the clinical presentations of the two diseases are very similar. In each province, one existing surveillance hospital site was selected and began screening individuals who met the dengue case definition for ZIKV, starting 15 February 2016. Later, surveillance for ZIKV was extended to four hospitals in the northern regions, six hospitals in the central coast regions and four hospitals in the central highlands region. All participating sites collected blood and urine samples for ZIKV testing from inpatients and outpatients who met the dengue case definition. Laboratory testing was performed on more than 2000 specimens from the south and central regions between May and August 2016. An additional 221 dengue-negative specimens that were collected by the dengue surveillance system in 2015 were tested for ZIKV to detect historical infections. Two cases of ZIKV were identified in the contemporaneous blood samples, while none was identified in the historical samples.

Between May and August 2016, in addition to the two ZIKV cases identified by the sentinel surveillance system, (*2*) four cases were identified by the WHO Event Management System (EMS) in travellers who developed symptoms returning home from Viet Nam. (*3*) In response to these cases, public health workers were deployed to the areas where the six case-patients either lived or travelled to search for other possible cases. Intensive mosquito control efforts, including reduction of mosquito breeding grounds, were carried out in these areas.

The Government of Viet Nam was able to increase capacity for surveillance and response through its collaborations and partnerships with WHO, CDC and other organizations and its commitment to the GHSA. GHSA was launched in 2014 as a collaboration between multiple institutions and nations with the aim to improve countries’ abilities to respond to public health emergencies. (*4*, *5*) One of the early GHSA investments in Viet Nam was the creation of a national EOC in Hanoi. The national EOC was able to receive, analyse, interpret and share information in real time with national, regional and international partners during the ZIKV response. Partnerships between Vietnamese public health responders and outside organizations, including WHO and CDC, provided training opportunities for laboratorians and epidemiologists. Laboratorians from the four RPHIs attended a CDC ZIKV laboratory training workshop in China, Taiwan, China, while on-the-ground epidemiology training was provided by international experts. In addition, resources such as primer/probe sequences and positive control RNAs were shared from outside institutions. Together, these collaborations and partnerships allowed Viet Nam to rapidly respond to the ZIKV threat.

The ZIKV PHEIC allowed Viet Nam to test its newly enhanced response system and identify areas that needed to be modified or expanded. Several lessons were learnt. First, while the GDPM led surveillance efforts at the national level, the mode of implementation was determined at the regional level. This practice led to variations in surveillance strategies in different regions, making it challenging to relate the data. In future responses, it would be useful to create a unified implementation plan for surveillance that could be consistently applied throughout the country. Second, while co-opting an existing surveillance system meant a new surveillance system could be established rapidly, it resulted in the creation of an inadequate response. In this response, the dengue surveillance system was initially used as the base for ZIKV surveillance. The dengue system was focused on inpatient surveillance. This turned out to be a poor fit for ZIKV surveillance given that most ZIKV patients have a mild clinical course. Finally, although a system for analysing and visualizing data was available at the national level, surveillance data were recorded and reported manually at the district and regional levels, resulting in a significant delay between data collection and data analysis and reporting.

Currently the VMoH is working to improve data accessibility by creating data warehouses to integrate data sources and building three additional regional EOCs. All EOCs will be networked and be able to collect, analyse, display and share data in real time. The networked data warehouses at the EOCs will integrate data from notifiable diseases and sentinel surveillance systems, as well as from laboratory and immunization databases. These advances are being supported through the GHSA and by collaborating partners. It is expected that these continued improvements will greatly facilitate future responses to emerging threats in Viet Nam.
